# Evaluation of changes to the *Rickettsia rickettsii* transcriptome during mammalian infection

**DOI:** 10.1371/journal.pone.0182290

**Published:** 2017-08-23

**Authors:** Sean P. Riley, Ludovic Pruneau, Juan J. Martinez

**Affiliations:** Vector-Borne Disease Laboratories, Department of Pathobiological Sciences, Louisiana State University School of Veterinary Medicine, Baton Rouge, Louisiana, United States of America; University of Arkansas for Medical Sciences, UNITED STATES

## Abstract

The lifecycle of *Rickettsia rickettsii* includes infection of both mammalian and arthropod hosts, with each environment presenting distinct challenges to survival. As such, these pathogens likely have distinctive transcriptional strategies for infection of each host. Herein, we report the utilization of next generation sequencing (RNAseq) and bioinformatic analysis techniques to examine the global transcriptional profile of *R*. *rickettsii* within an infected animal, and to compare that data to transcription in tissue culture. The results demonstrate substantial *R*. *rickettsii* transcriptional alteration *in vivo*, such that the bacteria are considerably altered from cell culture. Identification of significant transcriptional changes and validation of RNAseq by quantitative PCR are described with particular emphasis on known antigens and suspected virulence factors. Together, these results suggest that transcriptional regulation of a distinct cohort of genes may contribute to successful mammalian infection.

## Introduction

*Rickettsia rickettsii* is the causative agent of Rocky Mountain spotted fever (RMSF), the most severe tick transmitted infection in the western hemisphere with human case fatality rates comparable to the most dangerous of all North American bacterial diseases [1]. This bacterium survives in nature through an endozoonotic cycle of infection of mammalian and tick hosts. As such, the bacteria are faced with diverse challenges for infection of and transition between individual hosts. This feature of zoonotic infection suggests that the bacteria have different pathogenic strategies for each host, and, therefore, likely experience significant transcriptional variations throughout the life cycle. This concept is supported in other bacterial pathogens whereby synchronization of gene expression is critical for successful infection [2]. Herein, we examine the hypothesis that *R*. *rickettsii* undergoes distinct transcriptional changes *in vivo* in a mammalian model of infection by comparing the global transcriptome of bacteria from laboratory culture and during fatal infection of *M*. *musculus*.

*Rickettsia* species transcriptional profiles have been examined in response to temperature changes, iron starvation, and time in culture [3–9]. These studies demonstrated a limited response to temperature change, but significant transcriptional changes when *R*. *rickettsii* are exposed to a blood meal while within the transmitting tick [7]. In depth examination of *R*. *rickettsii* within the tick established that the genes encoding for the Type IV Secretion System (T4SS), potentially secreted proteins, iron acquisition proteins, and surface exposed proteins are all regulated in response to blood. In contrast, analysis of *R*. *conorii* eschar samples indicated a significant decrease in translation machinery and upregulation of transcripts associated with stress [10]. This *in vivo* analysis of *R*. *conorii* differs from the current study in the method of *Rickettsia* transmission (tick versus needle inoculation) and tissue source (eschar versus morbidly infected liver). Additionally, the authors note that *R*. *conorii* in eschars may be undergoing host immune clearance in the inoculation eschar [10]. Together, these transcriptomic studies establish that *Rickettsia* species, and particularly *R*. *rickettsii*, have the ability to regulate transcription in response to environmental variations.

When developing an experimental strategy to expand upon these previous transcriptional analyses, we considered multiple material sources. Our first goal was to characterize the *R*. *rickettsii* transcriptome within the infected mammal; therefore, we chose to analyze *R*. *rickettsii* within the liver of moribund C3H/HeN mice, because this sample contains multiple infected host cells types with the largest rickettsial load currently established [11]. The highest ratio of *R*. *rickettsii* to *M*. *musculus* RNA was predicted to produce the highest number of sequencing reads that could be attributed to the bacteria, and thus the most complete analysis of rickettsial transcription within an infected animal.

The *in vivo* transcriptome data necessarily needed to be compared to another condition to identify transcriptional changes. There are multiple *in vitro* culture conditions that could serve as a comparison to *in vivo* samples. After considering multiple conditions, we reasoned that the best comparison would be to *R*. *rickettsii* from infected Vero cells. This condition was chosen because much of the current understanding of *R*. *rickettsii* biology was generated through Vero cell culture. First, Ellison et.al. [5] describes a global transcriptional analysis of Vero cell propagated *R*. *rickettsii* under different environmental conditions. By utilizing Vero cell cultured *R*. *rickettsii* in the current experimental setup, we would be able to more accurately compare our findings to that published data. Additionally, Ammerman et.al. [12] is the current standard for *in vitro* propagation of *R*. *rickettsii*. We reasoned that most past and future *in vitro* propagations would utilize this culture method, so this RNA would be the most viable for comparison to *in vivo* transcription.

Transcriptional analysis of surface-exposed and secreted proteins of obligate intracellular *Rickettsia* are of particular importance because of the essential functions mediated by these proteins, including host cell invasion, escape from the phagolysosome, intracellular motility, and manipulation of the host response to infection [13–16]. Experiments designed to identify surface proteins as defined by exposure to surface biotinylation have identified approximately 70 likely surface exposed rickettsial proteins [9, 17]. Of these proteins, the surface cell antigen (Sca) family of autotransporters have been individually localized to the bacterial surface [9, 18–21]. Secretion of Sca proteins is dependent upon the Sec-dependent Type V Secretion, but *Rickettsia* also encode for Sec-Tol, Tat, Type I, and Type IV Secretion Systems [22]. As such, *R*. *rickettsii* is likely to encode for a plethora of surface and secreted proteins, and demonstration of transcriptional regulation of these potential virulence factors will aid future analyses of *R*. *rickettsii* pathogenesis.

Herein, we utilized next-generation sequencing technologies to compare quantities of and changes to *R*. *rickettsii* RNA *in vivo* during mouse infection and *in vitro* during tissue culture. The transcriptional alterations observed *in vivo* indicate that the bacterium is actively adapting to the mammalian host, and that our current understanding of *in vitro R*. *rickettsii* RNA and/or protein content is different than is found within the infected animal. We report changes to genes encoding for rickettsial surface proteins, Type IV Secretion System, and ribosomal subunits. The results of this study will serve as a basis for analyzing the pathobiology of *R*. *rickettsii* infection within the mammalian host.

## Materials and methods

### Cell lines and RNA samples

African green monkey kidney cells (Vero, ATCC) were cultured and maintained under standard conditions as described previously [19]. Briefly, 150cm^2^ flasks of confluent Vero cells were infected at 1 bacteria per host cell. The bacteria were allowed to infect at 34°C 5%CO_2_ for five days with the media being changed at day 3. *R*. *rickettsii* were purified from Vero cells by needle lysis and centrifugation over a sucrose cushion [12]. Total RNA was isolated twice from two separate *R*. *rickettsii* Sheila Smith propagations for a total of four replicates (2 technical replicates for each of 2 biological replicates). These samples are subsequently referred to as *“in vitro*.*”* Similarly, total RNA was isolated from liver tissues of C3H/HeN mice at five days post infection with 1x10^7^ intravenous *R*. *rickettsii* [11]. These mice were moribund and were euthanized prior to succumbing to *R*. *rickettsii* infection. RNA was isolated twice from two separate livers for a total of four replicates (2 technical replicates for each of 2 biological replicates). These samples will subsequently be referred to as “*in vivo*.”

### RNA isolation, DNAse treatment, and enrichment of prokaryotic mRNA

Total RNA was isolated from the eight total samples using Purelink RNA Mini Kit (Ambion). DNA was removed from the RNA purification using Ambion Turbo DNAse according to manufacturer’s instructions. Removal of DNA contamination was verified by *sca1* PCR as previously described [11]. Quality control was performed on a Fragment Analyzer (Advanced Analytical) to determine the RNA quality number (RQN). All RNA samples had a RQN > 7.6 (7.6–9.8). After confirmation of RNA structural integrity, 5μg RNA was subjected to MicrobeExpress and MicrobEnrich (Invirogen) protocols to remove bacterial rRNA and mammalian RNA, respectively.

### cDNA synthesis and sequencing

cDNA libraries were constructed using the Ion Total RNA-Seq Kit v2 (Life). Templates for RNASeq were prepared with the Ion PI Hi-Q OT2 200 Kit (Life) using the Ion OneTouch 2 System for Ion Proton System semiconductor sequencing. These templates were sequenced using the Ion PI Hi-QSequencing 200 Kit and Ion PI Chip v2, on the Ion Proton Sequencer. Preliminary analysis of the ensuing sequencing data pertaining to quality of the run, read lengths, and coverage was performed using the Torrent Suite software. Total reads, usable reads, and mean read length for each sample are indicated in S1 Table.

### Sequence analysis

The sequence reads from the four in vivo samples were first scanned for adapters and quality filtered using cutadapt with Partek Flow [23]. Next, reads from *Rickettsia rickettsii* were identified using a modification of the two-step method, whereby samples were first downloaded from NCBI on 08/2016, then aligned to *Mus musculus* GRCm38.p4 with tophat2, then the unaligned fastq reads were aligned to *R*. *rickettsii* Sheila Smith (NC_009882.1), using TMAP[24]. The four *in vitro* samples were aligned to the *R*. *rickettsia* genome using the SeqManNGEN portion of DNASTAR [25]. To determine the number of reads that were mapped to *R*. *rickettsii* genes, htseq was used to determine gene counts. The htseq counts for each gene were converted to RPKM using the formula: (number of reads)/[(gene size/1,000)x(total reads/1,000,000)] [26], and these values were averaged for the four *in vivo* sequencing reactions. DESeq2 was used for differential gene expression analysis by contrasting the *in vitro* and *in vivo* groups [27]. DEseq utilizes an adjusted value of significance (p_adj_) which is described in “Statistical analyses.” A summary of the analysis pipeline is diagramed in S1 Fig.

### qRT-PCR validation

*R*. *rickettsii* gDNA loads in mouse organs were quantified by TaqMan quantitative PCR (qPCR). Total chromosomal DNA from liver tissue (*in vivo*) and purified *R*. *rickettsii* (*in* vitro) was purified using PureLink Genomic DNA mini kit (Invitrogen) and normalized to 50μg/ml. TaqMan qPCR reactions were run as previously described [11] on the LightCycler 480 II system (Roche). PCR reactions were run as follows: 58°C for 3min, 95°C for 10min, followed by 50 cycles of 95°C for 15s and 58°C for 60s. Standard curves were generated using the plasmids pEC3 (*sca1*) and pYC82 (*actin*). gDNA content in organs are expressed as the number of *R*. *rickettsii sca1* copies per murine *M*. *musculus* or *C*. *aethiops actin* gene copies.

Changes to the transcriptional content of specific genes were determined by quantitative reverse transcription PCR (qRT-PCR). 200ng of DNA-fee RNA was subjected to Superscript Vilo (Invitrogen) reverse transcription. The quantity of each cDNA was determined by qPCR using the primers described in S2 Table. PCR reactions were run as follows: 95°C for 10 min, followed by 50 cycles of 95°C for 30s, 63°C for 30s, and 72°C for 60s followed by melting curve. *R*. *rickettsii* gDNA was used for calculation of standard curve. qPCR-derived fold change values are expressed as:

$$\mathrm{fold}\;\mathrm{change}=\log_{2}\left[\frac{\left(\frac{{\mathrm{cDNA}}_{in\;vivo}}{{\mathrm{gDNA}}_{sca1\;in\;vivo}/{\mathrm{gDNA}}_{actin\;in\;vivo}}\right)}{\left(\frac{{\mathrm{cDNA}}_{in\;vitro}}{{\mathrm{gDNA}}_{sca1\;in\;vitro}/{\mathrm{gDNA}}_{actin\;in\;vitro}}\right)}\right]$$

### Identification of open reading frames with signal sequences

To identify sec-dependent signal peptides the *R*. *rickettsii* protein reference sequence (GCF_000018225.1_ASM1822v1) was input into the signal sequence prediction algorithms: 1) Phobius [28] 2) PRED-TAT [29] 3) SignalP4.1 [30] and 4) LipoP [31]. Proteins categorized as “Secreted Proteins” were identified by 3 of the 4 servers (S3 Table).

### Sequence deposition

The 8 sequence output files and metadata are available from NCBI Sequence Read Archive under the accession number PRJNA382417. A summary of all RNAseq data is found in S4 Table.

### Animal research

Animal experiments were conducted in accordance with protocols approved by the Institutional Biological and Recombinant DNA Safety Committee (IBRDSC) and Institutional Animal Care and Use Committee (IACUC) at the Louisiana State University School of Veterinary Medicine. The University has a file with the Office of Laboratory Animal Welfare (OLAW), an approved Assurance Statement (#A3612-01).

### Statistical analyses

Correlation between qPCR and RNAseq results was performed by Shapiro-Wilk Test for Normality and Pearson analysis of correlation in Graphpad Prism. “p_adj_” values for fold change significance are computed in the DESeq2 package. This value uses the used Benjamin-Hochberg correction method. P_adj_ values of <0.01 are considered significant [32].

## Results

### RNAseq

The central hypothesis considered herein is that *R*. *rickettsii* utilizes genetic regulation to employ a specific transcriptional program for infection of the mammalian host. Accordingly, the experimental objective was to query *R*. *rickettsii* for transcriptional differences between bacteria derived from common cell culture and within the infected animal, called “*in vitro”* and *“in vivo”* respectively. To generate *in vitro* RNA samples, we purified *R*. *rickettsii* Sheila Smith from routine Vero cell culture at the time of maximum rickettsial burden. Bacteria at this stage of *in vitro* culture were the inoculum source for infection of mammals, and can therefore be considered at pre-infection transcriptional state. Additionally, transcriptional responses of *R*. *rickettsii* to environmental stimuli in Vero cell culture have been examined previously [5].

As a comparison, we purified *in vivo* whole RNA from within *R*. *rickettsii* infected *M*. *musculus* liver at the point where the animals were considered mortally infected [11]. This sample was chosen because it represents the highest rickettsial burden throughout mouse infection. This high bacterial burden would produce the highest ratio of *Rickettsia* to host RNA for technical purposes, and would also represent the highest number of input *Rickettsia* for the most heterogeneous *R*. *rickettsii* population. We performed four total RNA extractions on two different biological samples for each experimental group. These eight total RNA samples were subjected to reverse transcription and deep sequencing to determine the quantity and arrangement of RNA in each sample.

The most immediate challenge in analyzing the transcriptome of obligate intracellular bacteria is the pervasiveness of host RNA in the *in vivo* environment. This overabundance of host transcript masks the available *Rickettsia*-specific sequencing information. In order to overcome this obstacle without artificially amplifying rickettsial RNA, we used RNAseq with extensive bioinformatics analysis. This technology is based on direct cDNA sequencing and allows the detection and quantification of RNA from very small amounts of cellular materials [33]. Importantly, this method does not rely on non-specific amplification of cDNA, as is required to get sufficient cDNA for chip-based *Rickettsia* transcriptomics [8]. To maximize the prevalence of rickettsial mRNA we first performed depletion of eukaryotic RNA and bacterial ribosomal RNA before reverse transcription and deep sequencing. Of the 94–78 million reads per sample, the majority were discarded during quality control, leaving approximately 15 million usable output reads. Even after depletion of eukaryotic RNA, only 0.81–6.68% of *in vivo* sequencing reads mapped to the *R*. *rickettsii* genome. Reads that mapped to rRNA and tRNA were not analyzed, thereby leaving an average coverage depth of 19–128 reads per gene of the *R*. *rickettsii* protein coding sequence. As such, the results described herein represent a vigorous assessment of the transcriptional program of *R*. *rickettsii* within the infected mammal.

The 1345 predicted protein-encoding open reading frames of *R*. *rickettsii* Sheila Smith were analyzed for total *in vivo* cDNA quantity and expression changes. The average expression value of individual genes was calculated for the four *in vivo* samples, and is expressed as average reads per kilobase per million reads (RPKM), a value that normalizes for gene length. Expression changes to individual genes were calculated in the DEseq2 software package, and are expressed as fold change of *in vivo* samples as compared to *in vitro*. Fold change values are associated with an adjusted value of statistical significance (p_adj_) where p<0.01 is considered significant. In all, 235 genes were significantly upregulated *in vivo* and 264 genes were downregulated *in vivo*. These values represent 17% upregulated genes and 20% downregulated genes, thus demonstrating substantial transcriptional differences between *in vitro* and *in vivo Rickettsia rickettsii*.

### Validation of RNAseq by quantitative RT-PCR

**Fig 1:**
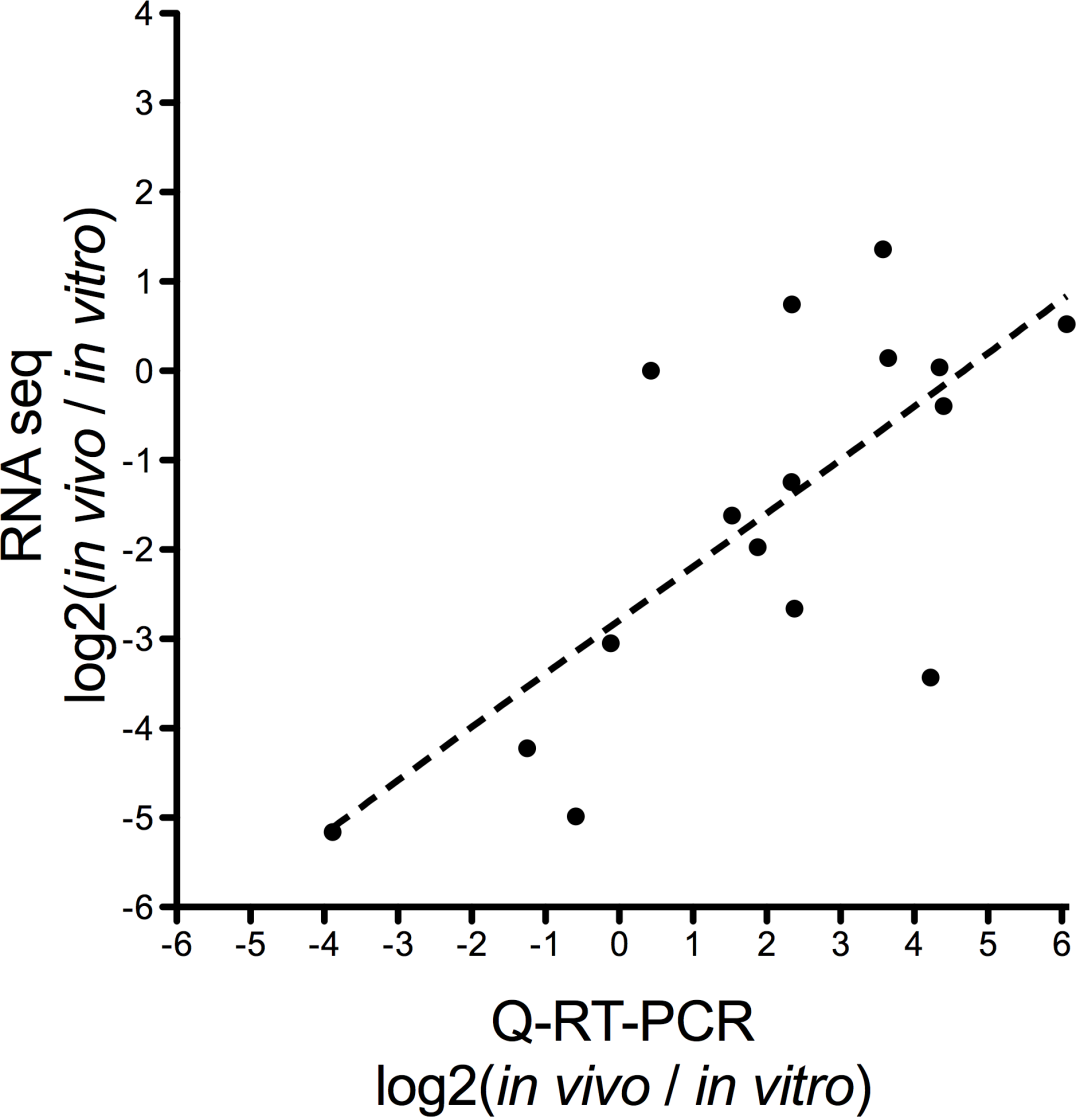
Comparison of transcriptional fold changes as determined by RNAseq and quantitative RT-PCR. *In vivo / in vitro* fold change values as determined by RNAseq (Y-axis) and qRT-PCR (X-axis). Pearson analysis of correlation p = 0.0015.

16 genes were chosen for individual analysis in order to validate the RNAseq data by an independent experimental method. To this aim, both RNA and gDNA were re-isolated from the RNAseq source material. The isolated RNA samples were subjected to DNAase treatment, reverse transcription, and quantitative PCR (qRT-PCR) to determine cDNA concentration for each of the 16 genes. In order to control for rickettsial nucleotide concentration in the PCR reaction, we utilized gDNA copies present in the biological sample. The concentration of *R*. *rickettsii sca1* copies per murine *actin* gDNA was determined in each sample, with a *sca1/actin* ratio of 3.27 for the *in vivo* infected liver sample and 78.32 in the *in vitro* sample. By adding equal amounts of cDNA and dividing the calculated cDNA concentration by the gDNA ratio, we are able to properly control for changes to the quantity of input *Rickettsia* RNA. This data transformation allows for accurate determination of the fold change in expression of each of the tested genes despite the contaminating host RNA. The qRT-PCR data for the 16 analyzed genes was compared to the fold change values obtained by RNAseq (Fig 1). Logically, if the two data acquisition mechanisms produce consistent results, then the two data sets should have a strong linear correlation. The results from each quantification method were compared and demonstrate a significant correlation with p = 0.0015. With these consistent results, we can extrapolate that the RNAseq data faithfully identifies changes to transcription.

### Genes encoding for secreted proteins

As *R*. *rickettsii* is an obligate intracellular pathogen, many necessary pathogenic functions occur at the bacterial surface. While several functions have been attributed to surface proteins, nothing is known about changes to the bacterial surface during the *R*. *rickettsii* lifecycle. Accordingly, the *R*. *rickettsii* Sheila Smith genome was queried by four signal sequence prediction algorithms (LipoP, Phobius, PRED-TAT, SignalP4.1) for the presence of a secretion signal. In all, 59 genes were predicted by 3 or more of the prediction algorithms to possess a secretion signal. This analytical method identified genes encoding for the well-known surface proteins OmpA, OmpB, Sca1, Sca2, 17kDa antigen, Adr1, and Adr2, but failed to identify some proteins previously identified by surface biotinylation [17].

**Fig 2:**
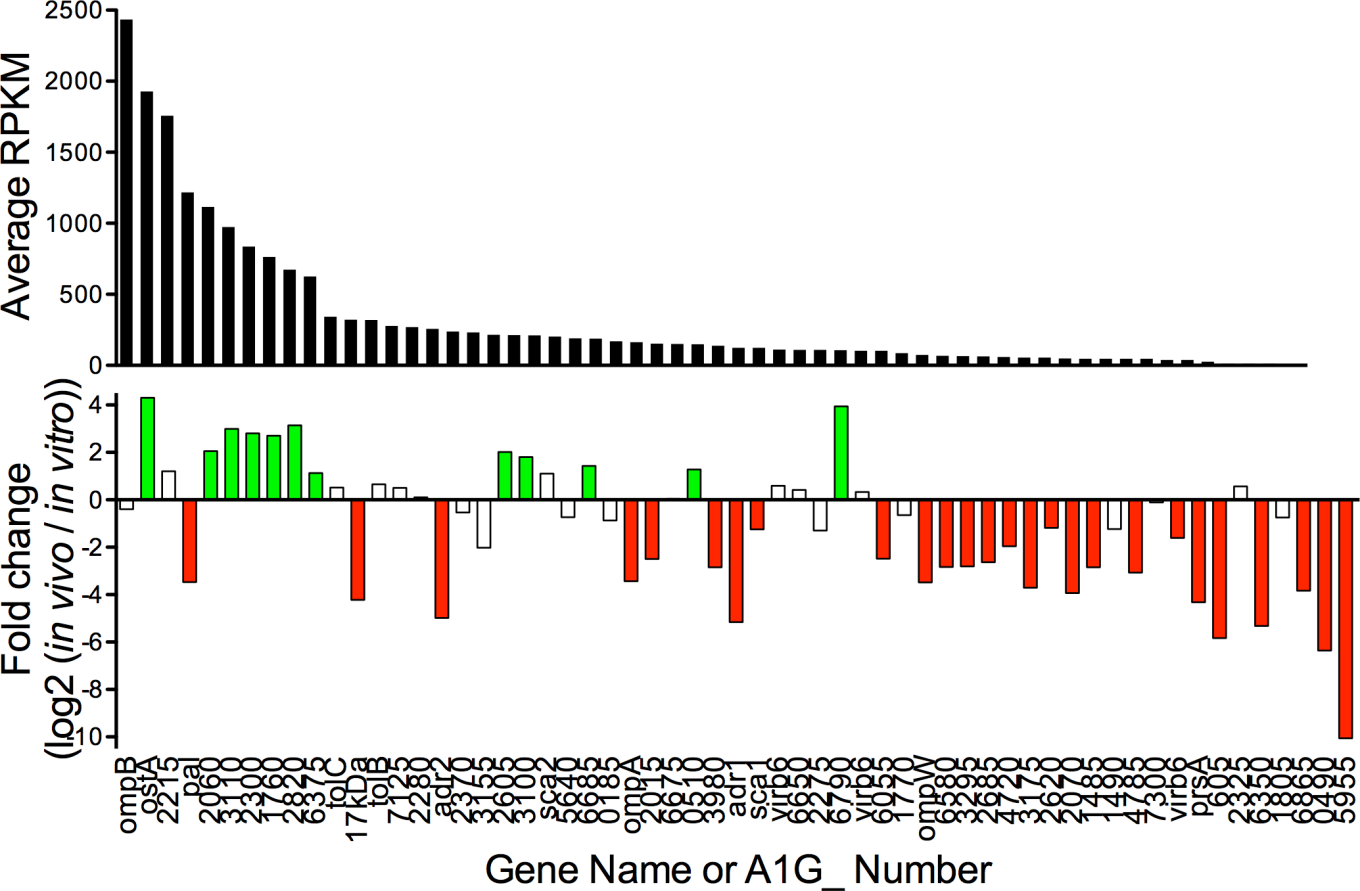
Analysis of *R*. *rickettsii* genes with predicted sec-dependent signal sequences. Four signal sequence prediction programs were utilized to identify 59 genes encoding for secreted or surface exposed *R*. *rickettsii* proteins. The quantity of transcript is expressed as average reads per kilobase per million reads (RPKM) for *in vivo* sequencing reactions. Additionally, each gene is graphed for transcriptional changes as expressed as fold change of *in vivo* over *in vitro*. Colored bars transcriptional change p_adj_<0.01.

The genes with predicted signal sequences were analyzed for *in vivo* RNA quantity (RPKM) and fold change of *in vivo* with relation to *in vitro* (Fig 2, S3 Table). In all, 12 genes were upregulated *in vivo* and 26 genes were significantly downregulated *in vivo*. As expected, the gene encoding for the ubiquitous surface protein *ompB* was the most frequently detected transcript, with high expression *in vivo* and insignificant expression change across the two conditions. By comparison, *ompA*, *sca1*, 17kDa antigen (A1G_07075), *adr1*, and *adr2* were all significantly downregulated *in vivo*. Upon identifying these differences in RNA content, we conclude that the antigen profile of *R*. *rickettsii in vivo* will likely be significantly different than has been observed for cultured bacteria. The most impressively upregulated gene, *ostA* (A1G_05645), encodes for an outer membrane barrel that contributes to LPS biosynthesis and inorganic solvent tolerance [34]. Interestingly, 9 of the 21 LPS genes involved in LPS biosynthesis and transport are significantly regulated (S5 Table), suggesting that *R*. *rickettsii* may alter the quantity or chemical structure of LPS.

**Fig 3:**
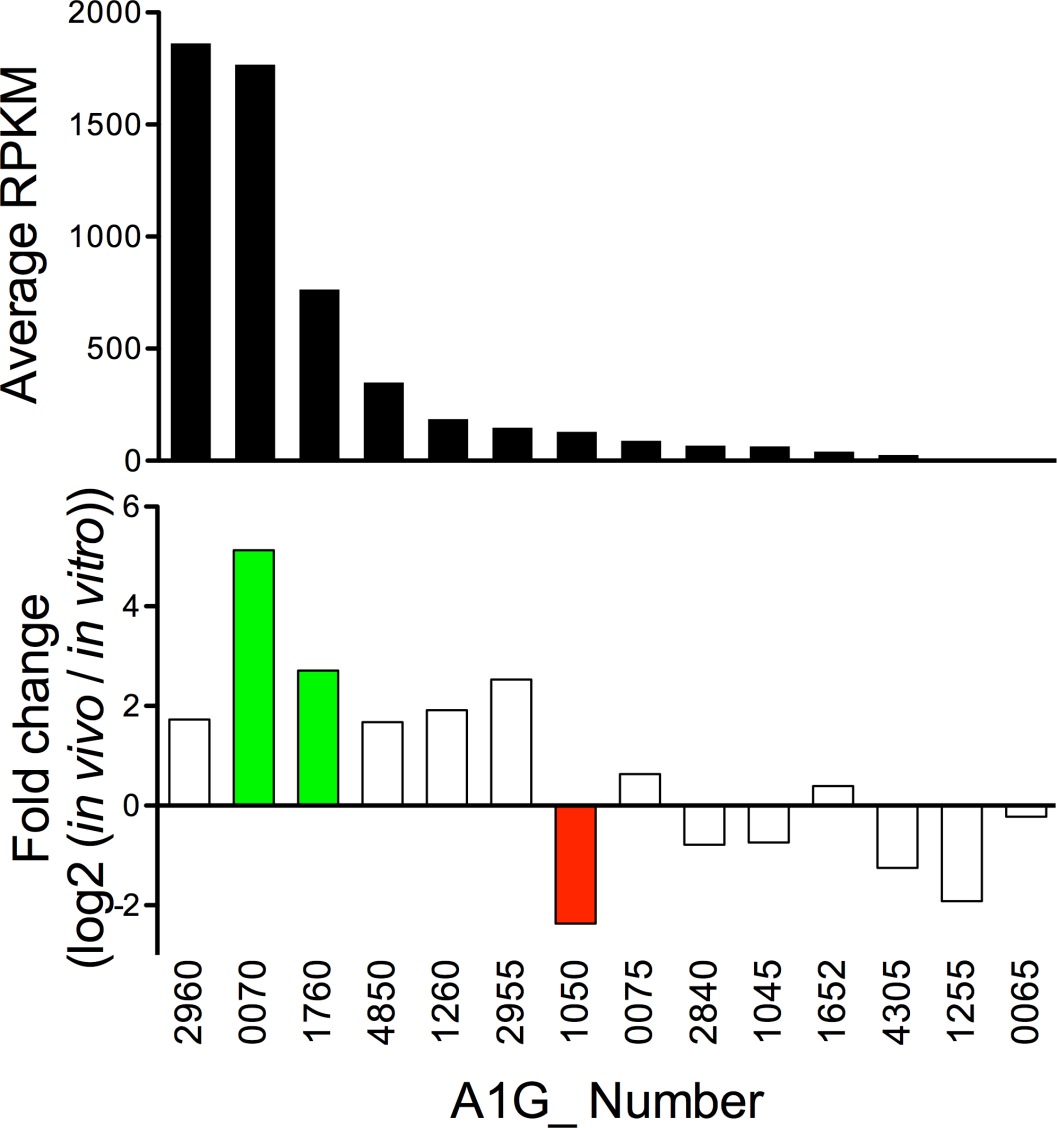
Changes to genes encoding for Ankyrin repeat (Ank) domain proteins. Transcription of 14 genes encoding for Ank domain proteins are analyzed for average *in vivo* expression (RPKM) and changes *in vivo* in relation to *in vitro*. Colored bars p_adj_<0.01.

*R*. *rickettsii* also encodes for 14 proteins containing Ankyrin repeat domains. The Ank domain containing proteins typically interact with eukaryotic proteins or DNA, and are therefore assumed to be secreted through Type I or IV Secretion Systems [35]. Indeed, Ank proteins from *Orientia tsutsugamushi* and *R*. *typhi* have been found within the host cytoplasm [36, 37]. The *R*. *rickettsii ank* genes were variably regulated (Fig 3, S5 Table). Analysis of total *in vivo* expression shows that both A1G_02960 and A1G_00070 were highly expressed (RPKM >1000). Conversely, A1G_01255 and A1G_00065 have extremely low expression *in vivo*.

### Type IV Secretion System

**Fig 4:**
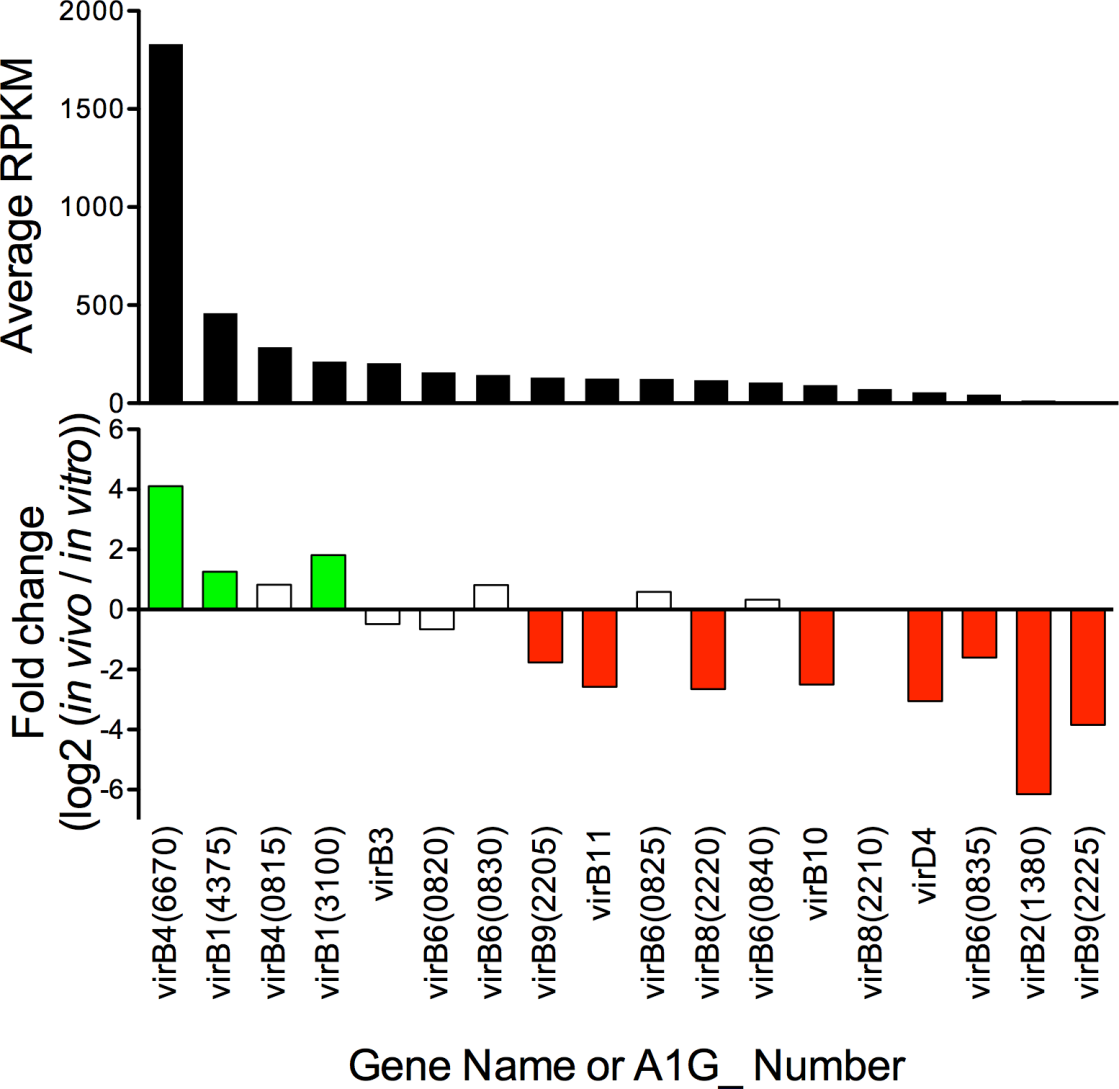
Analysis of genes encoding for Type IV Secretion System. The 18 annotated *virB* genes are analyzed for total *in vivo* transcription (RPKM) and changes to transcription *in vivo* as compared to *in vitro*. Colored bars p_adj_<0.01.

All Rickettsiales encode for a Type IV Secretion System (T4SS), consisting of a polypeptide complex that translocates proteins into the extracellular environment or directly into a host cell. Studies have established that T4SS are essential to pathogenesis in various bacterial species [38]. The genetic makeup of the *Rickettsia* T4SS has been thoroughly investigated, whereby the genetic complexity and conservation suggests that this system is essential for completion of the *Rickettsia* lifecycle [39–41]. Some of the genes encoding for T4SS are duplicated or scattered thereby producing many identically annotated genes [39]. Upregulation of the T4SS genes on one of the major operons in *R*. *rickettsii* was reported in the infected arthropod, while another major operon was downregulated [7]. In our case, the A1G_02205–02240 region (*virB9*, *virB8*, *virB9*, *virB10*, *virB11*, *virD11)* was significantly downregulated *in vivo* and A1G_00815–00840 was not significantly changed (Fig 4, S5 Table).

### *In vivo* suppression of genes encoding for ribosomal proteins

**Fig 5:**
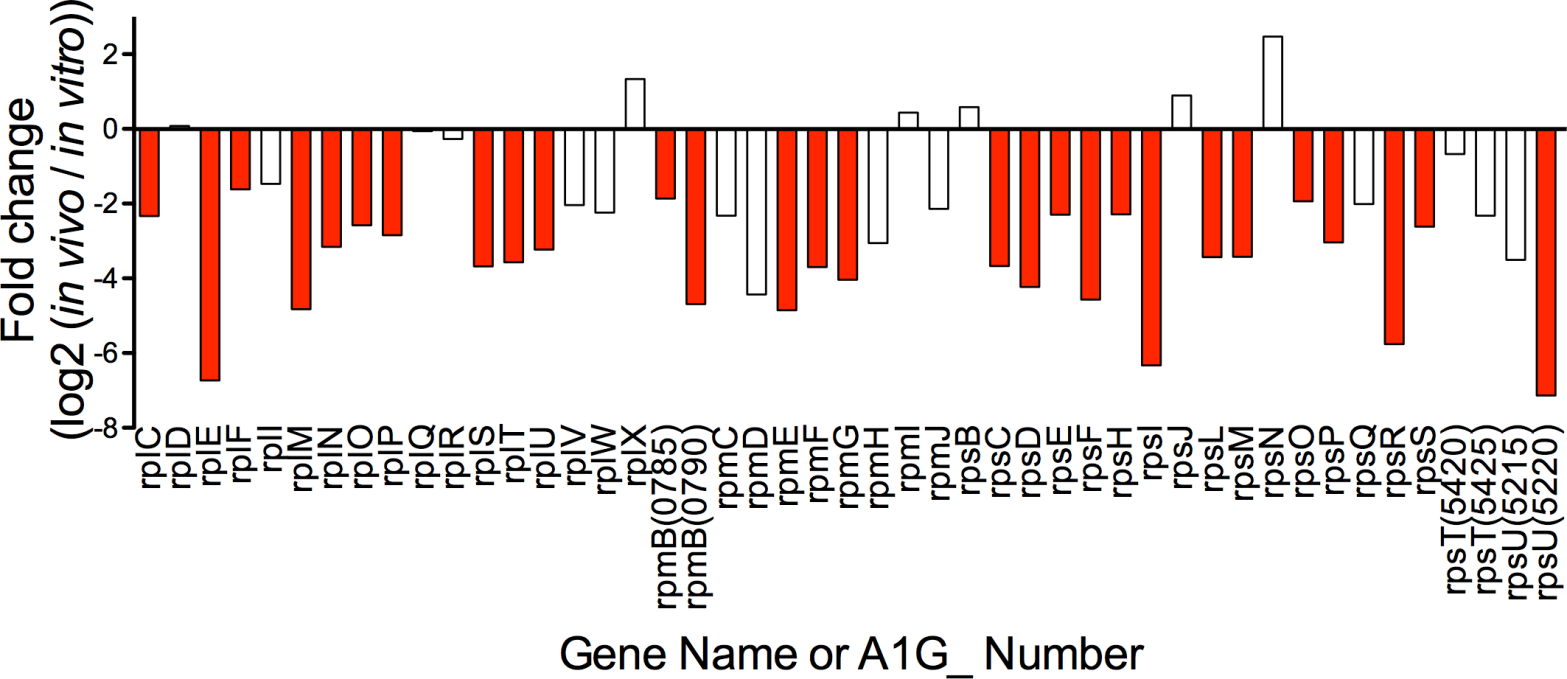
Downregulation of genes encoding for large (*rpl / rpm*) and small (*rps*) ribosomal subunits. Genes encoding for protein subunits of the large ribosomal subunit (*rpl*, *rpm)* and small ribosomal subunit (*rps*) graphed for changes in transcription *in vivo* in relation to *in vitro*. 28 of 47 genes are significantly repressed *in vivo*. Colored bars p_adj_<0.01.

Possibly the most conspicuous transcriptional change observed *in vivo* is the consistent and significant down regulation of the genes encoding for the large and small ribosomal subunits. It is pertinent to note that all RNA samples were treated to remove bacterial ribosomal RNA (rRNA) prior to sequencing. However, rRNA depletion does not affect the concentration of mRNA nucleotides that encode for the protein components of the ribosome. 28 of the 47 genes encoding for ribosomal subunit proteins (*rpl*, *rpm*, *rps*) were significantly downregulated, with no genes upregulated *in vivo* (Fig 5, S5 Table). This is a striking downregulation of the translational apparatus, and mirrors the results found in *R*. *conorii* inoculation eschars [10]. This finding suggests that *R*. *rickettsii* is potentially reducing its translational capacity *in vivo*, and is consistent with a stringent response [42].

## Discussion

Herein, we describe the use of RNAseq technology to ascertain quantity of and changes to *R*. *rickettsii* RNA content *in vivo* compared to *in vitro*. The *in vitro* RNA samples are prepared from *R*. *rickettsii* in routine Vero cell culture at the point of maximal bacterial burden. The transcriptome of those bacterial samples is compared to *in vivo R*. *rickettsii* derived from infected murine liver at the point of maximum morbidity. As such, comparison of these samples establishes adjustments made by the bacterium in response to the challenges of mammalian infection. The RNAseq data was validated by qRT-PCR to establish the validity of the transcriptomic data (Fig 1). RNAseq analysis of changes to the transcriptome demonstrated significantly altered transcription in 37% of the genes of the *R*. *rickettsii* genome. Importantly, we demonstrate changes to the quantity of transcripts encoding for secreted proteins (Fig 2), Ank domain containing proteins (Fig 3), the Type IV secretion apparatus (Fig 4), and translation machinery (Fig 5). Together, these results demonstrate that *R*. *rickettsii* actively adapts to its environment, and the quantity of many known or potential virulence factors are significantly changed *in vivo*.

The high bacterial load in the liver in this mouse model of *R*. *rickettsii* infection is different than occurs in the well-known guinea pig model of infection. *R*. *rickettsii-* associated liver pathology in human cases has been described in the past, with specific mention of mononuclear hepatitis [43–45]. Liver pathology has also been noted for infection with the closely related bacterium, *R*. *conorii* [46]. Additionally, rickettsial infection of cells of mononuclear and hepatocyte cells has been extensively examined in the past [47–49]. As such, the infected cell types and lesions observed in the mouse model of *R*. *rickettsii* infection are observed in human infections.

Since the vast majority of analyses into the molecular mechanisms of obligate intracellular bacteria pathogenesis are performed in a tissue culture setting, a relevant question pertains to the makeup of the bacteria within the infected animal. Herein, we establish that the transcriptional makeup of the bacteria *in vivo* is different than has been traditionally analyzed *in vitro*. An example of this phenomenon is described in Fig 2 where many genes encoding for secreted and surface proteins are regulated *in vivo*. This group of proteins includes the defined antigens on the *R*. *rickettsii* surface, including OmpA and OmpB [50–53]. The importance of defining *in vivo* expression of these surface proteins is exemplified by complications stemming from vaccination against the *Borrelia burgdorferi* OspA antigen which is entirely repressed within the infected mammal [54–56]. Of the major *R*. *rickettsii* surface antigens, *ompA*, *sca1*, *adr1*, *adr2*, and 17kDa antigen (A1G_07075) are all downregulated *in vivo* (Fig 2, S3 Table). However, despite decreased *in vivo* expression, there is substantial transcript present for all of these antigens, suggesting that targeting the encoded proteins still has potential for preventative efficacy. In contrast, the most prevalent transcript encoding for secreted proteins is the major surface antigen *ompB*. This gene is expressed at very high levels both *in vivo* and *in vitro*, and therefore remains a primary target for future attempts at preventative vaccination. Based on the expression levels demonstrated in Fig 2, there are additional genes with high *in vivo* expression levels (>1000 RPKM), including *ostA*, A1G_02215, *pal*, and A1G_02060. We posit that these and other highly expressed secreted proteins merit further investigation into the potential for contributing to *R*. *rickettsii* pathogenesis and for advancing efforts at preventative therapeutics.

Many of the *R*. *rickettsii* genes that catalyze the biosynthesis or transport of lipopolysaccharide (LPS) are actively regulated (S5 Table). Regulation of the quantity or makeup of LPS in mammalian pathogens is well documented [57]. In fact, the intracellular bacterium *Coxiella burnetii* actively regulates LPS content as an immune evasion strategy [58–60]. *R*. *rickettsii* contains a polysaccharide-rich electron-translucent region surrounding the bacterial membrane [61]. This LPS-rich area has been found to vary in its physical properties, but the source of this variation is unknown. As such, the transcriptional changes in LPS biosynthesis and transport suggests that *R*. *rickettsii* LPS structure may vary between cell culture, within the infected mammal, and in arthropod hosts [62]. Exploration of the consequences of this phenomenon may illuminate rickettsial pathobiology.

The *R*. *rickettsii* Ank domain containing proteins are also presumed to be secreted. Multiple roles for Ank proteins within the mammalian host cytoplasm have been defined [35]. The *R*. *rickettsii ank* genes are variably regulated with no discernable expression pattern (Fig 3). However, A1G_01760 is a particularly interesting gene, because it encodes for a *Rickettsia* ankyrin-repeat protein 1 (RARP-1) homolog with a predicted N-terminal Sec secretion signal and C-terminal Ank domain [63]. The very high level of *in vivo* transcription (average RPKM = 763) suggests that this protein is readily transcribed/translated, and should be found in abundance outside of the host cell. A1G_02960 and A1G_00070 are also upregulated and highly expressed *in vivo*, but lack a probable Sec secretion signal. These high levels of expression suggest a significant *in vivo* function if these two proteins are secreted into the host cell.

All *Rickettsia* species encode for a Type IV Secretion System (T4SS). T4SS have been demonstrated to be essential in various other pathogens [38]. The genes encoding for the *Rickettsia rickettsii* T4SS macromolecular structure (*virB*) are found in a series of scattered genetic loci with multiple gene duplications [40]. There are two major loci encoding for VirB proteins, and the locus containing *virB4* and five *vir6* genes is not regulated (Fig 4). In contrast, the *virB9*, *virB8*, *virB9*, *virB10*, *virB11*, *virD11* (A1G_02205–02240) locus is significantly repressed *in vivo* (Fig 4). These findings suggest that the transmembrane Type IV secretion apparatus is present, but may be underrepresented within the infected mammal.

As demonstrated in Fig 5, genes encoding for translational machinery are significantly downregulated *in vivo*. It is again important to note that rRNA depletion has no effect on the presence of mRNA encoding for ribosomal proteins. The downregulation of genes encoding for ribosomal proteins is similar to a previous report describing translation machinery downregulation *R*. *conorii* within inoculation eschars [10]. Though *R*. *conorii* eschar sample and *R*. *rickettsii* liver samples are quite different, both analyses reached the same conclusion, thereby suggesting that this may be a widespread phenomenon.

In contrast to the shared observation of ribosomal mRNA downregulation, *in vivo* analysis of *R*. *conorii* eschar samples and *R*. *rickettsii* liver produced quite different results. The upregulated transcripts reported in *R*. *conorii* did not correlate with the present study. 22 of the 31 reported upregulated *R*. *conorii* transcripts could be definitively assigned to a *R*. *rickettsii* homolog. Only 14 of the 22 shared genes were upregulated in *in vivo* in both *R*. *conorii* and *R*. *rickettsii*. Taken together, these results suggest that while both *in vivo* analyses found a decrease in translational machinery, the *R*. *conorii* global transcriptome in an inoculation eschar differs from *R*. *rickettsii* in the infected liver. This is not unexpected as *Rickettsia* in human eschar and murine liver are experiencing different challenges. Though *Rickettsia* are frequently cultured from inoculation eschars [64–66], it is clear that these bacteria are located in a zone of limited fluid perfusion within significantly necrotic dermis and epidermis. In contrast, liver derived *R*. *rickettsii* are infecting a heterogeneous population of mononuclear cells (Kupffer cells), endothelial cells, and hepatocytes [11]. As such, care must be taken when comparing the only two transcriptional analysis of *Rickettsia* species in a mammal.

In summary, we have utilized RNAseq technologies to define the *in vivo* transcriptome of *R*. *rickettsii*, the causative agent of Rocky Mountain spotted fever. The global RNA profile *in vivo* is vastly different than is found in routine tissue culture. This data further advances the suitability of those genes that are highly expressed or upregulated *in vivo* as research topics. We have described regulation of RNAs encoding for secreted proteins, LPS production, Type IV secretion, and translational machinery. The observed regulation correlates with our hypothesis that *R*. *rickettsii* utilizes transcriptional alterations during pathogenesis in the mammalian host. Investigation into the molecular mechanisms of transcriptional regulation will greatly increase our understanding of the pathogenesis of these severe human infections, and experimental disruption of regulation may contribute to development of interruptive therapeutics.

## Supporting information

S1 Fig Diagram of programs used to analyze RNAseq reads to determine transcriptional changes. (DOCX)

S1 Table Summary of quantity, quality, and length of sequencing reads prior to bioinformatic analysis. (DOCX)

S2 Table Genes targeted for qPCR validation, primer sequences, and calculated fold changes (log_2_(*in vivo / in vitro*)). (DOCX)

S3 Table RNA content (RPKM), transcriptional changes, and signal sequence prediction results. (DOCX)

S4 Table *In vivo* expression (RPKM), fold change (log_2_(*in vivo / in vitro*)), and significance (p_adj_) for all *R. rickettsii* protein encoding genes. (XLSX)

S5 Table RNA content and transcriptional changes of genes encoding for LPS biosynthesis/transport, Ank-domain containing proteins, Type IV Secretion System, and ribosomal proteins. (DOCX)
