# Supplementary figures and images for: Evaluation of changes to the Rickettsia rickettsii transcriptome during mammalian infection
Source: PLoS One. 2017 Aug 23;12(8):e0182290. doi: 10.1371/journal.pone.0182290 (PMC5568294; doi:10.1371/journal.pone.0182290)

**S1 Fig.** Diagram of programs used to analyze RNAseq reads to determine transcriptional changes.


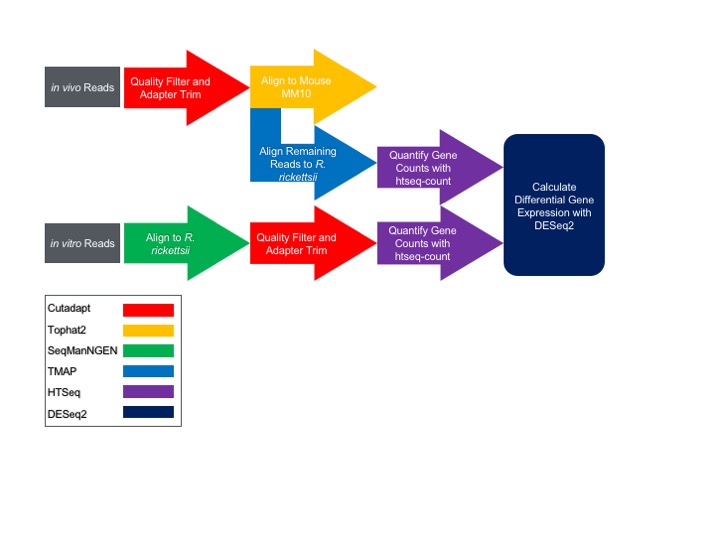

Supplement: S1 Fig — (DOCX) [file pone.0182290.s001.docx]
