# Supplementary material for: Evaluation of changes to the Rickettsia rickettsii transcriptome during mammalian infection
Source: PLoS One. 2017 Aug 23;12(8):e0182290. doi: 10.1371/journal.pone.0182290 (PMC5568294; doi:10.1371/journal.pone.0182290)
Supplement: S1 Table — (DOCX) [file pone.0182290.s002.docx]

**S1 Table.** Summary of quantity, quality, and length of sequencing reads prior to bioinformatic analysis.

| Sample Name | Total Reads | Usable Reads | Mean Read Length |
| --- | --- | --- | --- |
| *in vitro* 1 | 94,319,714 | 67% | 110bp |
| *in vitro* 2 | 85,241,695 | 63% | 112bp |
| *in vitro* 3 | 86,905,511 | 70% | 128bp |
| *in vitro* 4 | 86,494,109 | 65% | 110bp |
| *in vivo* 1 | 89,732,273 | 64% | 91bp |
| *in vivo* 2 | 91,914,263 | 67% | 129bp |
| *in vivo* 3 | 92,064,562 | 67% | 93bp |
| *in vivo* 4 | 77,985,044 | 59% | 111bp |
