# Supplementary material for: Evaluation of changes to the Rickettsia rickettsii transcriptome during mammalian infection
Source: PLoS One. 2017 Aug 23;12(8):e0182290. doi: 10.1371/journal.pone.0182290 (PMC5568294; doi:10.1371/journal.pone.0182290)
Supplement: S2 Table — (DOCX) [file pone.0182290.s003.docx]

**S2 Table.** Genes targeted for qPCR validation, primer sequences, and calculated fold changes (log_2_(*in vivo* / *in vitro*)).

| Gene I.D. | Primer Name | Primer Sequence | Product Size (b.p.) | qPCR Fold Change |
| --- | --- | --- | --- | --- |
| A1G_06030 | *ompB* F | AAA CGT AAC TCG GCA GCA TG | 170 | 4.40 |
|  | *ompB* R | TGC TAG CAA TCC CGG TAA CA |  |  |
| A1G_06990 | *ompA* F | AAC CGA CAG TAC TGC TGG TT | 122 | 4.22 |
|  | *ompA* R | CCG GTG ATG AGA CAG CAG TA |  |  |
| A1G_07045 | *adr1* F | CAA AAG CAG CAC CAA CAC CT | 225 | -3.89 |
|  | *adr1* R | GAG ATC GGT GCG GGT TAC TA |  |  |
| A1G_07050 | *adr2* F | CTA ATG CAG GAC CAA CAC CG | 155 | -0.59 |
|  | *adr2* R | AAT CCG GAG CTG CGA CTA AT |  |  |
| A1G_00640 | *ompW* F | AAC GGC TGC AAG GGA TGT AT | 111 | -0.43 |
|  | *ompW* R | CGG TTA TGG AGG TGA TGC GT |  |  |
| A1G_01745 | *tolC* F | TCC TGA GGG AGG AGC TCA AT | 179 | 6.07 |
|  | *tolC* R | TGA GCA GCA TCC ACT CCT TG |  |  |
| A1G_03170 | *murG* F | TTT GGC GGT AGT CAA GGA GC | 117 | 4.34 |
|  | *murG* R | CAT CTA ATG CCG CTT GCT GG |  |  |
| A1G_01910 | *ddl* F | TCT TTG TCC TGC TCC GCT AC | 198 | -0.11 |
|  | *ddl* R | AGC TGC AAT TTC CGG CAC TA |  |  |
| A1G_03040 | *dacF* F | TGC TGA GGG CAT GAA AGG TT | 136 | 1.53 |
|  | *dacF* R | CTC TTG CGG TAG TTT GCT GC |  |  |
| A1G_00020 | *rfbA* F | TCG GCG GTA TTC CTT TGT TGG | 135 | 3.65 |
|  | *rfbA* R | TGC CCT AAA CTA TCC GCA ACA |  |  |
| A1G_03280 | *pheS* F | ATG ATC ATC CCG CAA GGC AA | 186 | 2.34 |
|  | *pheS* R | GGC GTA TGC GTC ATA TCC GA |  |  |
| A1G_01480 | *uspA* F | CAT AAA CGG CAC CGC TAC CTA | 199 | 1.88 |
|  | *uspA* R | TGC GAC GAA CTT TCA AGC AAA |  |  |
| A1G_06490 | *recA* F | TGG GTG ATG CTC AAA TGG CT | 160 | 2.37 |
|  | *recA* R | ACC GCC TGT CGT AGT TTC AG |  |  |
| A1G_07075 | 17kDa F | GGC AAA GGA CAG CTT GTT GG | 169 | -1.25 |
|  | 17kDa R | TCC GGA TTA CGC CAT TCT ACG |  |  |
| A1G_04780 | *uvrC* F | CCG CCT CTT CAA AAC GCA TC | 152 | -3.58 |
|  | *uvrC* R | ATG CTC CTT GCG TCG GTA AA |  |  |
| A1G_00130 | *sca1* F | CTA CCG CTC CTT GGA ATG TTA | 208 | 2.33 |
|  | *sca1* R | CAA GCT CGT TAT TAC CCC GAA |  |  |
