# Supplementary material for: Evaluation of changes to the Rickettsia rickettsii transcriptome during mammalian infection
Source: PLoS One. 2017 Aug 23;12(8):e0182290. doi: 10.1371/journal.pone.0182290 (PMC5568294; doi:10.1371/journal.pone.0182290)
Supplement: S3 Table — (DOCX) [file pone.0182290.s004.docx]

**S3 Table.** RNA content (RPKM), transcriptional changes, and signal sequence prediction results.

| Gene and/or (A1G_) | Average *in vivo* RPKM | Fold Change  log_2_(*in vivo / in vitro*) | padj | LipoP | Phobius | PRED-TAT | SignalP |
| --- | --- | --- | --- | --- | --- | --- | --- |
| *ompB* | 2435 | -0.40 | 0.71 | + | + |  | + |
| *ostA* | 1928 | 4.30 | 2.43E-10 | + | + | + |  |
| 2215 | 1757 | 1.21 | 0.037 | + | + | + |  |
| *pal* | 1217 | -3.47 | 3.67E-49 | + | + | + |  |
| 2060 | 1116 | 2.05 | 9.42E-05 | + | + | + | + |
| 3110 | 973 | 2.99 | 3.46E-06 | + | + |  | + |
| 2300 | 837 | 2.81 | 1.87E-12 | + | + | + |  |
| 1760 | 763 | 2.71 | 2.86E-10 |  | + | + | + |
| 2820 | 673 | 3.15 | 1.11E-05 | + | + |  | + |
| 6375 | 625 | 1.13 | 2.20E-07 | + | + | + |  |
| *tolC* | 343 | 0.52 | 0.15 | + | + | + | + |
| 17kDa | 322 | -4.22 | 3.95E-25 | + | + | + | + |
| *tolB* | 320 | 0.66 | 0.060 | + | + | + |  |
| 7125 | 279 | 0.51 | 0.24 | + | + | + | + |
| 2280 | 269 | 0.11 | 0.87 | + | + | + |  |
| *adr2* | 257 | -4.98 | 7.30E-28 | + | + | + | + |
| 2370 | 238 | -0.53 | 0.55 |  | + | + | + |
| 3155 | 232 | -2.013 | 0.017 | + | + | + |  |
| 2605 | 216 | 2.02 | 5.74E-09 | + | + | + |  |
| 3100 | 213 | 1.81 | 0.00035 | + | + |  | + |
| *sca2* | 209 | 1.11 | 0.10 | + | + | + | + |
| 5640 | 203 | -0.73 | 0.12 | + | + |  | + |
| 6685 | 190 | 1.43 | 0.0084 | + | + | + |  |
| 0185 | 189 | -0.87 | 0.019 | + | + | + |  |
| *ompA* | 170 | -3.43 | 4.71E-99 | + | + | + |  |
| 2015 | 164 | -2.50 | 0.0042 |  | + | + | + |
| 6675 | 154 | 0.044 | 0.924 | + | + | + | + |
| 0510 | 151 | 1.28 | 0.047 | + | + | + |  |
| 3980 | 148 | -2.84 | 0.00044 | + | + | + |  |
| *adr1* | 139 | -5.16 | 3.23E-59 | + | + | + | + |
| *sca1* | 125 | -1.24 | 6.42E-06 | + | + | + |  |
| *virb6*(0825) | 124 | 0.59 | 0.18 | + | + |  | + |
| 6650 | 111 | 0.42 | 0.49 | + | + | + |  |
| 2275 | 109 | -1.29 | 0.014 | + | + | + |  |
| 6790 | 109 | 3.94 | 0.0063 | + | + | + |  |
| *virb6*(0840) | 106 | 0.33 | 0.35 | + | + |  | + |
| 6055 | 104 | -2.48 | 2.71E-05 | + | + |  | + |
| 1770 | 103 | -0.64 | 0.14 |  | + | + | + |
| *ompW* | 86 | -3.48 | 4.81E-08 | + | + |  | + |
| 6580 | 71 | -2.83 | 0.0083 | + | + | + |  |
| 3295 | 67 | -2.80 | 0.0085 | + | + | + | + |
| 2685 | 66 | -2.63 | 2.02E-07 | + | + | + |  |
| 4720 | 64 | -1.96 | 2.39E-06 | + | + | + |  |
| 3175 | 59 | -3.701 | 1.18E-06 | + | + | + |  |
| 2620 | 54 | -1.18 | 0.030 | + | + | + | + |
| 2070 | 54 | -3.93 | 1.52E-05 |  | + | + | + |
| 1485 | 48 | -2.84 | 9.02E-06 | + | + | + | + |
| 1490 | 47 | -1.23 | 0.12 | + | + | + |  |
| 4785 | 47 | -3.07 | 0.0037 | + | + | + | + |
| 7300 | 46 | -0.11 | 0.92 | + | + |  | + |
| *virb6*(0835) | 45 | -1.60 | 0.00013 | + | + | + | + |
| *prsA* | 37 | -4.32 | 1.72E-19 | + | + | + | + |
| 605 | 36 | -5.83 | 1.66E-28 | + | + |  | + |
| 2325 | 26 | 0.57 | 0.65 | + | + | + |  |
| 6350 | 12 | -5.32 | 4.60E-05 | + | + | + | + |
| 1805 | 11 | -0.75 | 0.66 | + | + | + |  |
| 6865 | 11 | -3.834 | 0.00020 | + | + | + |  |
| 0490 | 0 | -6.36 | 0.000774 | + | + |  | + |
| 5955 | 0 | -10.05 | 2.96E-10 | + | + |  | + |
