# Supplementary material for: Evaluation of changes to the Rickettsia rickettsii transcriptome during mammalian infection
Source: PLoS One. 2017 Aug 23;12(8):e0182290. doi: 10.1371/journal.pone.0182290 (PMC5568294; doi:10.1371/journal.pone.0182290)
Supplement: S5 Table — (DOCX) [file pone.0182290.s006.docx]

**S5 Table.** RNA content and transcriptional changes of genes encoding for LPS biosynthesis/transport, Ank-domain containing proteins, Type IV Secretion System, and Ribosomal proteins.

| Gene and/or (A1G_) | Average *in vivo* RPKM | Fold Change  (log_2_(*in vivo / in vitro*)) | padj |
| --- | --- | --- | --- |
| **LPS Biosynthesis and Transport** | | | |
| *ostA / lptD* | 1928 | 4.30 | 2.43E-10 |
| *lpxD* | 1033 | 1.49 | 0.085 |
| *lptG* | 466 | 2.27 | 0.056 |
| *lpxK* | 422 | 4.62 | 4.20E-06 |
| 5860 | 351 | 1.56 | 0.071 |
| *rfaJ* | 268 | 7.04 | 0.00018 |
| *lptB* | 234 | 0.16 | 0.81 |
| *rfbA* | 221 | 0.14 | 0.88 |
| *kpsF* | 190 | 1.59 | 0.012 |
| *lpxB* | 129 | 0.26 | 0.63 |
| *lpxL* | 108 | 0.27 | 0.74 |
| *rfbE* | 107 | -2.41 | 0.0010 |
| 7035 | 100 | 3.09 | 3.27E-05 |
| 2975 | 100 | -1.28 | 0.019 |
| *kdtA* | 98 | 2.51 | 1.04E-05 |
| *kdsA* | 70 | -1.80 | 0.0035 |
| *lpxA* | 65 | -0.73 | 0.19 |
| *lptF* | 61 | 0.50 | 0.55 |
| *lpxC* | 41 | -4.11 | 5.09E-06 |
| 2760 | 35 | 0.38 | 0.71 |
| 5855 | 7 | -1.46 | 0.21 |
| **Type IV Secretion System** | | | |
| *virB4*(6670) | 1830 | 4.11 | 1.25E-05 |
| *virB1*(4375) | 459 | 1.26 | 0.0006 |
| *virB4*(0815) | 285 | 0.82 | 0.041 |
| *virB1*(3100) | 213 | 1.81 | 0.00035 |
| *virB3* | 204 | -0.48 | 0.61 |
| *virB6*(0820) | 157 | -0.66 | 0.044 |
| *virB6*(0830) | 145 | 0.81 | 0.21 |
| **Type IV Secretion System (Continued)** | | | |
| *virB9*(2205) | 132 | -1.76 | 0.0021 |
| *virB11* | 126 | -2.57 | 3.12E-14 |
| *virB6*(0825) | 124 | 0.59 | 0.18 |
| *virB8*(2220) | 118 | -2.66 | 0.00077 |
| *virb6*(0840) | 106 | 0.33 | 0.35 |
| *virB10* | 92 | -2.50 | 6.16E-06 |
| *virB8*(2210) | 73 | 0.00 | 1.00 |
| *virD4* | 56 | -3.05 | 8.54E-10 |
| *virB6*(0835) | 45 | -1.60 | 0.00013 |
| *virB2*(1380) | 14 | -6.15 | 3.11E-10 |
| *virB9*(2225) | 9 | -3.84 | 0.00033 |
| **Ank Domain-Containing** | | | |
| 2960 | 1862 | 1.73 | 0.12 |
| 0070 | 1767 | 5.13 | 9.05E-10 |
| 1760 | 763 | 2.71 | 2.86E-10 |
| 4850 | 349 | 1.67 | 0.064 |
| 1260 | 186 | 1.92 | 0.035 |
| 2955 | 148 | 2.53 | 0.077 |
| 1050 | 128 | -2.36 | 1.95E-08 |
| 0075 | 89 | 0.63 | 0.52 |
| 2840 | 67 | -0.78 | 0.38 |
| 1045 | 64 | -0.74 | 0.29 |
| 1652 | 40 | 0.39 | 0.75 |
| 4305 | 27 | -1.25 | 0.064 |
| 1255 | 6 | -1.92 | 0.21 |
| 0065 | 0 | -0.22 | NA |
| **Ribosomal Subunits** | | | |
| *rplC* | 123 | -2.33 | 0.0019 |
| *rplD* | 487 | 0.07 | 0.87 |
| *rplE* | 0 | -6.73 | 0.0003 |
| *rplF* | 137 | -1.6 | 0.003 |
| *rplI* | 242 | -1.47 | 0.011 |
| *rplM* | 114 | -4.83 | 2.46E-15 |
| *rplN* | 100 | -3.15 | 0.0004 |
| *rplO* | 94 | -2.58 | 9.45E-05 |
| *rplP* | 20 | -2.84 | 0.001 |
| *rplQ* | 767 | -0.05 | 0.95 |
| *rplR* | 359 | -0.27 | 0.57 |
| **Ribosomal Subunits (Continued)** | | | |
| *rplS* | 77 | -3.68 | 1.03E-07 |
| *rplT* | 68 | -3.56 | 0.0009 |
| *rplU* | 229 | -3.23 | 1.21E-06 |
| *rplV* | 100 | -2.04 | 0.038 |
| *rplW* | 58 | -2.24 | 0.019 |
| *rplX* | 1788 | 1.34 | 0.21 |
| *rpmB*(0785) | 0 | -1.86 | N/A |
| *rpmB*(0790) | 25 | -4.69 | 1.92E-08 |
| *rpmC* | 10 | -2.32 | 0.12 |
| *rpmD* | 0 | -4.43 | 0.028 |
| *rpmE* | 143 | -4.85 | 1.18E-06 |
| *rpmF* | 60 | -3.69 | 0.006 |
| *rpmG* | 70 | -4.04 | 0.002 |
| *rpmH* | 0 | -3.05 | 0.16 |
| *rpmI* | 289 | 0.44 | 0.65 |
| *rpmJ* | 95 | -2.149 | 0.15 |
| *rpsB* | 865 | 0.58 | 0.55 |
| *rpsC* | 37 | -3.67 | 0.0004 |
| *rpsD* | 152 | -4.22 | 1.70E-13 |
| *rpsE* | 243 | -2.29 | 2.19E-05 |
| *rpsF* | 6 | -4.56 | 0.0005 |
| *rpsH* | 47 | -2.28 | 0.006 |
| *rpsI* | 5 | -6.33 | 2.19E-07 |
| *rpsJ* | 1235 | 0.89 | 0.24 |
| *rpsL* | 36 | -3.43 | 0.001 |
| *rpsM* | 139 | -3.43 | 5.62E-11 |
| *rpsN* | 5806 | 2.48 | 0.099 |
| *rpsO* | 160 | -1.93 | 0.006 |
| *rpsP* | 46 | -3.03 | 1.81E-06 |
| *rpsQ* | 79 | -2.01 | 0.013 |
| *rpsR* | 0 | -5.76 | 0.003 |
| *rpsS* | 51 | -2.62 | 0.015 |
| *rpsT*(5420) | 182 | -0.67 | 0.70 |
| *rpsT*(5425) | 62 | -2.32 | 0.012 |
| *rpsU*(5215) | 133 | -3.51 | 0.014 |
| *rpsU*(5220) | 0 | -7.13 | 8.19E-05 |
